# *In vitro* comparison of anthelmintic efficacy across *Gyrodactylus* species

**DOI:** 10.17221/79/2025-VETMED

**Published:** 2026-04-30

**Authors:** Marketa Ondrackova, Jitka Kolarova, Katerina Skocovska

**Affiliations:** ^1^Institute of Vertebrate Biology of the Czech Academy of Sciences, Brno, Czech Republic; ^2^South Bohemian Research Centre of Aquaculture and Biodiversity of Hydrocenoses, Faculty of Fisheries and Protection of Waters, University of South Bohemia in České Budějovice, Vodňany, Czech Republic

**Keywords:** fenbendazole, gyrodactylids, ivermectin, levamisole, survival

## Abstract

Gyrodactylosis, caused by monogenean parasites of the genus *Gyrodactylus*, poses a significant threat to aquaculture, particularly under intensive farming conditions. This study evaluated the *in* *vitro* effectiveness of three veterinary anthelmintics – fenbendazole (FBZ), levamisole hydrochloride (LHC), and ivermectin (IVM) – against four *Gyrodactylus* species collected from wild cyprinid hosts. Parasite survival was monitored over 48 h at 6–7 °C. LHC showed the highest efficacy at both tested concentrations (30 and 50 mg/l), achieving near-complete mortality within 8 hours. FBZ and IVM were significantly less effective, with 23% and 33% of parasites surviving, respectively. Species-specific differences in drug susceptibility were observed, particularly with FBZ and IVM, with *G. carassii* showing consistently higher sensitivity than *G. laevis*, *G. rutilensis*, and *G. vimbi*. Lower water temperatures probably contributed to reduced drug efficacy and prolonged parasite survival. These findings highlight the importance of species-level evaluation and temperature considerations when selecting anthelmintic treatments for gyrodactylosis. *In* *vitro* testing under controlled conditions offers valuable insights into parasite susceptibility and complements *in* *vivo* approaches. Further research incorporating broader parasite diversity, temperature ranges, and pharmacological profiles is recommended to optimise treatment strategies in aquaculture.

Gyrodactylosis, caused by monogenean parasites of the genus *Gyrodactylus*, poses a significant threat, particularly to intensively farmed fish populations. While infections in natural environments are often self-limiting, the parasite’s direct life cycle and viviparous reproduction enable rapid proliferation under intensive aquaculture conditions, where high fish densities facilitate transmission (Buchmann and Bresciani 2006). Mechanical damage from the opisthaptor, combined with feeding on epithelial cells and mucus, compromises skin integrity, leading to secondary bacterial and fungal infections and, ultimately, high mortality rates (Bakke et al. 2007). Severe infections can cause osmotic imbalance and extensive tissue damage. For example, one of the most impactful gyrodactylid species, *Gyrodactylus salaris*, has caused devastating epidemics in wild Atlantic salmon (*Salmo salar*) in Norway and continues to threaten salmonid farming across Europe (Hansen et al. 2016).

Management of ectoparasitic infections in aquaculture, including gyrodactylosis, relies primarily on chemical therapeutics administered via short-term or long-term baths, and in some cases, oral dosing (Rigos et al. 2024). Short-term baths are commonly used to eliminate ectoparasites on the skin and gills and to allow replacement of the therapeutic solution with clean water post-treatment. Long-term treatments using lower concentrations of medicinal substances are applied to entire intensive farming systems, with subsequent gradual water changes. Oral administration, though less frequently employed, enables systemic drug delivery via medicated feed (Svobodova et al. 2007; Noga 2010; Palikova et al. 2019). Each method presents specific challenges in dosage control, host tolerance, and environmental impact.

Although over 99 compounds and treatment combinations have been tested against *Gyrodactylus* spp., few have been systematically compared under standardised conditions (Schelkle 2012), and no treatment has proven universally effective against gyrodactylids (Schelkle et al. 2015). Common agents such as formaldehyde, malachite green, rotenone, and aluminium salts show variable efficacy and raise concerns about ecological impact and human health risks (Rowland et al. 2006). Discarded residues of commercial anthelmintics can harm the environment by causing toxicity to non-target organisms and posing a risk of resistance development (Rigos et al. 2024). Alternative treatments such as herbal extracts and natural saponins show promising efficacy with reduced toxicity (e.g., Schelkle et al. 2013; Zhou et al. 2021), though practical implementation remains limited by cost and regulatory constraints.

For fish intended for human consumption, veterinary medicinal products (VMPs) approved for use in fish (EU Decree No. 470/2009) are preferred as first-line treatment. Pharmacologically active substances of VMPs must have established maximum residue limits (MRLs). Alternatively, veterinarians may prescribe drugs “off label” on their own responsibility in accordance with Decree No. 344/2008 Coll., using VMPs intended for other animal species (e.g., farmed mammals and poultry), other indications, or a human medicinal product, prepared according to the given requirements in a pharmacy according to a “magistraliter” prescription. Recommended “off-label” VMPs used for treatments of gyrodactylosis also include fenbendazole, levamisole, and ivermectin (Svobodova et al. 2007; Noga 2010; Palikova et al. 2019).

Benzimidazole fenbendazole (FBZ) is relatively safe but often shows low efficacy or narrow safety margins (Alves et al. 2019; Dong et al. 2025). FBZ has demonstrated high efficacy in goldfish when administered orally or via short-term baths, though prolonged exposure may cause oxidative stress and histopathological changes (Dong et al. 2025). Levamisole hydrochloride (LHC) is effective in specific cases (Schmahl and Taraschewski 1987; Kolarova et al. 2022a), but its use can alter haematological profile, plasma biochemistry, and antioxidant indicators, causing oxidative stress (Kolarova et al. 2022b). Ivermectin (IVM), an avermectin with broad antiparasitic activity, is approved for use in mammals intended for human consumption (cattle, sheep, pigs, and horses) (Kositz et al. 2022). Its antiparasitic effects in fish have been described by Santamarina et al. (1991) and Pike and Wadsworth (1999), but environmental toxicity from residues (Wang et al. 2020) significantly limits its use in aquaculture.

Species-specific differences in anthelmintic efficacy are well-documented in nematodes but rarely studied in monogeneans. Shirakashi et al. (2021) tested febantel against two gill monogeneans of the *Seriola* spp. but did not directly compare the parasite species. Alves et al. (2019) evaluated the efficacy of anthelmintics against four monogenean species of *Colossoma macropomum*, suggesting differential responses among parasite species. In nematode models, drug efficacy varied across strongylid species (Hu et al. 2013), and patterns in resistance to some anthelmintics explained parasite species-specific differences in susceptibility of cyathostome nematodes to treatment (Traversa et al. 2009) and in parasite intraspecific genetic diversity (Shaver et al. 2024). Similar patterns are expected in fish-parasite systems, where host physiology, immune response, and parasite biology interact to influence treatment outcomes.

The aim of this study was to test the efficacy of selected anthelmintic drugs in the form of VMPs against the causative agent of gyrodactylosis in fish under controlled* in vitro* conditions. At the same time, we evaluated species-specific sensitivity among different *Gyrodactylus* species.

## MATERIAL AND METHODS

### Ethical statement

This study was conducted in accordance with the ethical regulations of the Czech Republic and was approved by the Animal Care and Use Committee of the Institute of Vertebrate Biology CAS, Brno (Permit No. UBO-MO-17-01). The method of fish sampling, maintenance, and euthanasia procedures complied with national legislation, including § 7 of Act No. 114/1992 on the Protection of Nature and Landscape, and § 6, 7, 9 and 10 of Regulation No. 419/2012 on the Care, Breeding and Use of Experimental Animals.

### Collection of *Gyrodactylus* parasites

Wild fish were collected to obtain a broad range of *Gyrodactylus* species. Six fish species, including *Blicca bjoerkna*, *Gobio gobio*, *Rhodeus amarus*, *Rutilus rutilus*, *Scardinius erythrophthalmus*, and *Squalius cephalus*, were sampled by electrofishing in the Dyje River near the city of Břeclav during late autumn (water temperature ranging between 6.5 and 7.5 °C). Fish were transported to the outdoor tubs placed in the garden of the Institute of Vertebrate Biology, CAS, and maintained until dissection. Fish were killed by severing the spine and examined under a binocular microscope for the presence of *Gyrodactylus* spp. Collected worms were individually transferred to pre-prepared 12-well titration plates containing 2 ml of either a selected commercial drug or dechlorinated water (control) and kept at 6–7 °C to minimise temperature-induced stress.

### *In vitro* tests

**Figure 1:**
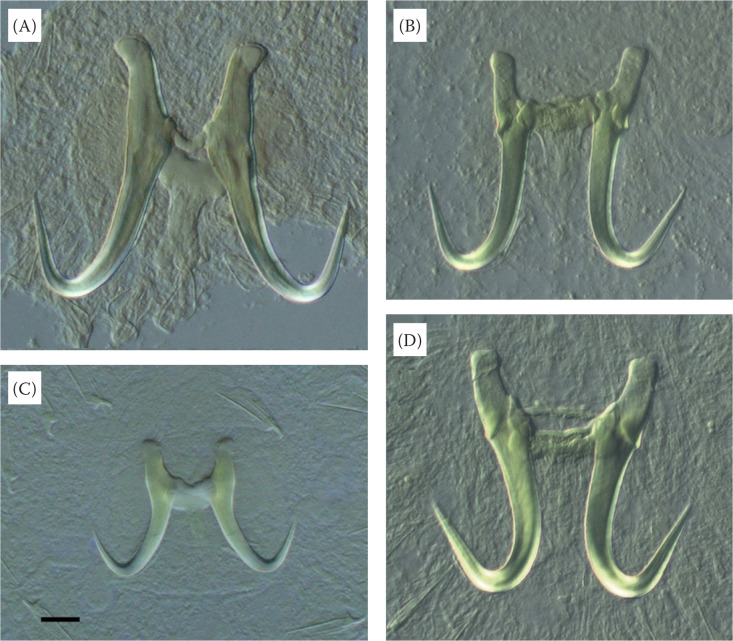
Photomicrographs of the haptoral hard parts of four *Gyrodactylus* species selected for *in* *vitro* testing of the sensitivity to selected anthelmintic drugs in the form of veterinary medicinal products, using Nomarski contrast (A) *Gyrodactylus carassii*; (B) *Gyrodactylus rutilensis*; (C) *Gyrodactylus laevis*; (D) *Gyrodactylus vimbi*. Scale bar = 10 μm

Parasite survival/mortality in titration plates was monitored continuously at intervals of 1, 2, 3, 4, 5, 6, 8, 10, 12, 24, 36, and 48 hours. Each plate was inspected under the binocular microscope for approximately 1–2 min to assess the parasite status (alive/dead). *Gyrodactylus* was considered dead if no movement (especially of marginal hooks) was observed for 10 s during routine inspection and another 10 s after the completion of the entire plate inspection. Dead specimens were removed, placed on the microscope slide and mounted in glycerine ammonium picrate (Malmberg 1970). Parasites were identified to species level using haptoral morphology and metric features (Gusev 1985) under an Olympus BX53 light microscope equipped with a StreamMotion image analysis system (Olympus Optical Co., Hamburg, Germany). In total, 370 parasites were used, with 2 592 recordings made. Of the twelve species identified, four were selected for further analyses based on sufficient sample size in at least three treatments, including control: *Gyrodactylus carassii*, *G. laevis*, *G. rutilensis*, and *G. vimbi* (Figure 1). The number of tested species at individual drug concentrations and in the control is shown in Table 1, totalling 311 parasites with 2 170 recordings. Other species that occurred less frequently and were not included in the analysis were *G. gasterostei*, *G. gobii*, *G. gracilihamatus*, *G. leucisci*, *G. longoacuminatus*, *G. prostae*, *G. rhodei*, and *G. scardiniensis*.

**Table 1:** Numbers of tested parasites of four *Gyrodactylus* species in selected anthelmintic VMPs and control (CTRL)

Active substance	FBZ		LHC 30	LHC 50		IVM	CTRL
VMP	panacur 25%		levamisole hydrochloride 80%			biomectin 1%	dechlorinated tap water
Concentration	25 mg/l		30 mg/l	50 mg/l		0.031 mg/l	
*Gyrodactylus carassii*	20		15	26		21	20
*Gyrodactylus rutilensis*	10		13	15		11	15
*Gyrodactylus vimbi*	11		1	20		6	19
*Gyrodactylus laevis*	2		27	17		19	23
Total	43		56	78		57	77

FBZ = fenbendazole; LHC 30 = levamisole 30 mg/l; LHC 50 = levamisole 50 mg/l; IVM = ivermectin

### Tested substances

To assess potential differences in treatment efficacy between different *Gyrodactylus* species, representatives of three anthelminthic drug classes were used: FBZ at a concentration of 25 mg/l (VMP Panacur 25% plv ad us. vet.; Intervet International B.V., Boxmeer, the Netherlands), LHC in two concentrations, i.e., 30 mg/l and 50 mg/l [VMP Levamisole hydrochloride 80% mg/g plv. (800 mg LHC in 1 g of product); KELA, Laboratoria n.v., Hoogstraten, Belgium], and IVM at a concentration of 0.031 mg/l (VMP Biomectin 1% inj. ad us. vet.; Bioveta a.s., Ivanovice na Hané, Czech Republic) (Table 1). The concentration of particular drugs was not regularly monitored due to logistical reasons. Drug concentrations were selected based on prior studies demonstrating antiparasitic effects and safety for fish during therapeutic baths (Lacey 1988; Santamarina et al. 1991; Martin and Robertson 2010; Noga 2010; Kolarova et al. 2022a; Kolarova et al. 2022b). Based on our preliminary study showing the efficacy of LHC at a lower concentration than the recommended 50 mg/l (Kolarova et al. 2022a), we also use LHC at 30 mg/l. Dechlorinated tap water served as the control (CTRL).

### Statistical analysis

Survival analysis was performed using Kaplan–Meier curves to visualise parasite mortality patterns across treatments and species. Differences in drug efficacy and species susceptibility were tested using the log-rank test. All statistical analyses were conducted in Statistica v14.0.0.15 (TIBCO Software Inc., USA).

## RESULTS

### Overall efficacy of tested drugs

**Figure 2:**
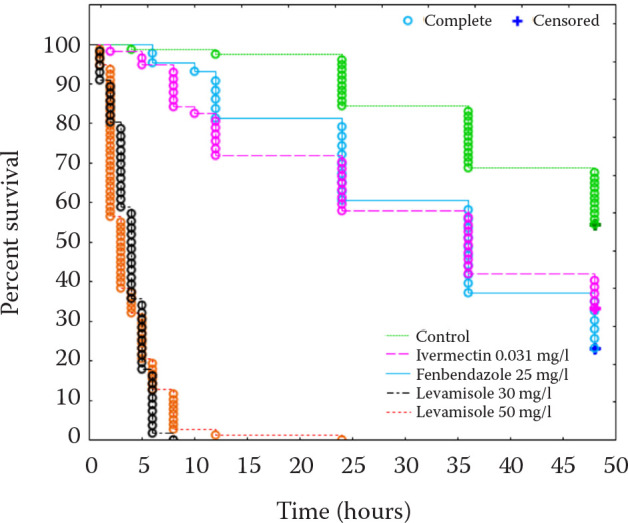
Kaplan–Meier plot comparing survival of all gyrodactylids together exposed to therapeutic baths with anthelmintic VMPs: fenbendazole 25 mg/l, levamisole 30 and 50 mg/l, ivermectin 0.031 mg/l and control (dechlorinated water) All therapeutic baths reduced *Gyrodactylus* survival compared to control (all *P* < 0.002), and this reduction was stronger in levamisole 30 and levamisole 50 compared to fenbendazole and ivermectin (*P* < 0.001)

All tested anthelmintic drugs significantly reduced the *in* *vitro* survival of *Gyrodactylus* spp. compared to the control within 48 h of treatment (Figure 2; χ^2^ = 226.9, d.f. = 4, *P* < 0.001; log-rank tests: all *P* < 0.002). Using a pooled dataset across *Gyrodactylus* spp., the most effective treatments were LHC at 30 mg/l and 50 mg/l, resulting in 100% and 97.5% mortality within 8 h, respectively. In contrast, treatments with IVM (0.031 mg/l) and FBZ (25 mg/l) were significantly less effective (Figure 2; all *P* < 0.001), with 33% and 23% of parasites surviving after 48 h, respectively. There was no significant difference in efficacy between IVM and FBZ treatments [*P* = 0.659; see Electronic Supplementary Material (ESM) Table S1].

When the species were analysed, similar trends were observed. Four *Gyrodactylus* species, namely *Gyrodactylus carassii*, *G. laevis*, *G. rutilensis* and *G. vimbi*, were tested (Figure 1, Table 1). LHC at both concentrations was highly effective across all parasite species, with comparable effects on parasite survival (*P* > 0.05; ESM Figure S1; Table S1), except for *G. carassii*, where LHC 50 mg/l was significantly more effective than LHC 30 mg/l (ESM Figure S1). Unlike pooled data, treatment by IVM did not differ from control in *G. laevis*, *G. rutilensis* and *G. vimbi*. Similarly, FBZ showed no significant effect in *G. rutilensis* (ESM Figure S1, Table S1).

### Species-specific susceptibility to treatment

**Figure 3:**
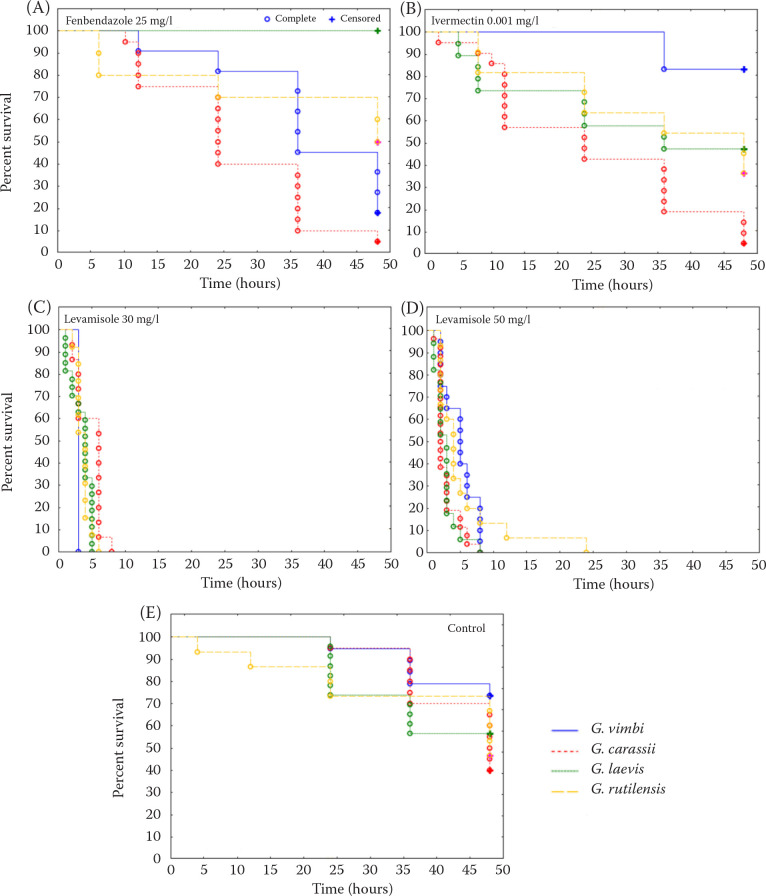
Kaplan–Meier plot showing *in vitro* survival of four *Gyrodactylus* species in four therapeutic treatments with anthelmintic veterinary medicinal products (A) Fenbendazole 25 mg/l; (B) ivermectin 0.031 mg/l; (C) levamisole 30 mg/l; (D) levamisole 50 mg/l, and (E) control in dechlorinated water, within 48 hours

No significant differences in survival were observed among the four *Gyrodactylus* species in the control group (Figure 3E; χ^2^ = 3.47, d.f. = 3, *P* = 0.321). However, significant interspecific differences emerged under treatment with less effective drugs, i.e., FBZ and IVM (χ² = 10.55 and 10.09, d.f. = 3, *P* = 0.014 and 0.018, respectively; Figure 3A,B). For FBZ,* G. carassii* showed the highest susceptibility, with 95% mortality within 48 hours.

Among the three species tested (excluding *G. laevis* due to low sample size), 50% of *G. rutilensis* and 18% of *G. vimbi* survived, compared to only 5% of *G. carassii* (log-rank tests: *P* = 0.011 and 0.021, respectively). A similar pattern was observed for IVM. *Gyrodactylus carassii* had significantly lower survival (5%) compared to *G. laevis* (48%, *P* = 0.026), *G. rutilensis* (37%, *P* = 0.034), and *G. vimbi* (83%, *P* = 0.002). However, results for *G. vimbi* should be interpreted cautiously due to the small sample size (*n* = 6).

Treatment with LHC showed less pronounced interspecific differences. No significant variation in parasite survival was found among species treated with LHC 30 mg/l (Figure 3C; χ^2^ = 5.67, d.f. = 3, *P* = 0.129), with all parasites dying within 5 h (*G. laevis*), 6 h (*G. rutilensis*) and 8 h (*G. carassii*). Efficacy in *G. vimbi* was not tested due to the low sample size (*n* = 1). In contrast, treatment with LHC 50 mg/l revealed significant differences across parasite species (Figure 3D; χ^2^ = 13.05, d.f. = 3, *P* = 0.005). While *G. carassii*, *G. laevis*, and *G. vimbi* reached 100% mortality within 6 h, two specimens of *G. rutilensis* survived for 12 and 24 hours. Survival of *G. carassii* and *G. laevis* was significantly lower than that of *G. rutilensis* and *G. vimbi* (all *P* < 0.05, ESM Table S1).

## DISCUSSION

The results of *in vitro* testing showed significant differences in the efficacy of individual anthelminthic drugs, regardless of parasite species. LHC demonstrated the highest efficacy at both concentrations (30 mg/l and 50 mg/l), while FBZ (25 mg/l) and IVM (0.031 mg/l) were significantly less effective. Susceptibility to individual drugs varied significantly among *Gyrodactylus* species, with the differences most pronounced for less effective treatments. This variability indicates that species identity may play an important role in selecting appropriate therapeutic agents.

All tested anthelmintics recommended for fish ectoparasite treatment, including FBZ, LHC, and IVM (Noga 2010; Alves et al. 2019), significantly reduced the survival of* Gyrodactylus* compared to the control. LHC proved to be the most effective anthelmintic at both tested concentrations: 50 mg/l, commonly recommended against monogeneans (Kolarova et al. 2022a), and 30 mg/l, which was equally effective at 6–7 °C. However, despite its high efficacy, 100% mortality was achieved only after a period longer than generally recommended for therapeutic baths (typically 2 hours). While all parasites died within 6 h in the LHC 30 mg/l bath, two individuals survived for 12 and 24 h in the LHC 50 mg/l bath, indicating considerable individual variability in parasite susceptibility to the treatment.

The potential use of IVM in fish has been tested against various monogenean parasites. Although the concentration used in this study (0.031 mg/l) was effective *in* *vitro* against *Gyrodactylus* spp. infecting rainbow trout *Oncorhynchus mykiss*, it was found to be highly toxic to fish (Santamarina et al. 1991), leading to elevated muscle superoxide dismutase activity compared to the controls (Kolarova et al. 2022b). A similar result was reported for IVM baths (200–350 mg/l) used against various monogenean species in *Colossoma macropomum* (Alves et al. 2019). FBZ has recently been recommended for antiparasitic treatment in aquaculture due to its efficacy and safety for fish hosts (Kolarova et al. 2022b; Dong et al. 2025), but *in vitro* tests showed significantly lower efficacy compared to LHC, with 23% of parasites surviving a 48-h bath. These results align with those of Tojo et al. (1992), who found FBZ to be less effective *in vitro* than *in vivo*: all parasites died within 12 h at concentrations of 1.5–25 mg/l, whereas no *Gyrodactylus* mortality was observed *in* *vitro*. However, *in* *vivo* tests typically assess the presence of parasites on the host and do not monitor detached parasites. For example, LHC is known to induce detachment of parasites from host tissues (Taraschewski et al. 1988), and IVM causes paralysis of the worms, also resulting in detachment (Collymore et al. 2014). While *in* *vivo* tests indicate parasite clearance from the host, *in* *vitro* tests allow direct assessment of parasite susceptibility to specific drugs. These differences highlight the importance of combining both approaches when evaluating drug efficacy.

The generally lower efficacy observed in this study compared to published data may primarily reflect the influence of temperature. Temperature can significantly affect the therapeutic outcomes of antiparasitic treatment in freshwater fish (Woo 2021). This study was deliberately conducted during the cold season, as many *Gyrodactylus* species prefer lower water temperatures (Chubb 1977). At approximately 6–7 °C, parasites survived therapeutic baths significantly longer than at higher temperatures reported in other studies, e.g., 15 °C in Santamarina et al. (1991) or 20 °C in Yang et al. (2025). Low temperatures increase the likelihood of *Gyrodactylus* survival outside the host (Bakke et al. 2007), as also observed in our control group, where more than half of the parasites survived 48 hours. Reduced efficacy at low temperature may result from slowed metabolism in both the parasite and the host (e.g., Lohmus and Bjorklund 2015), which affects drug pharmacokinetics (Li et al. 2012) and may delay the onset of action of active compounds (Rairat et al. 2022).

Differences in drug efficacy among parasite species are typically studied in nematodes and often involve intergeneric comparisons (e.g., Hu et al. 2013; Lecova et al. 2014; Shirakashi et al. 2021). Interspecific comparison of four *Gyrodactylus* species treated *in* *vitro* with four anthelmintic baths revealed significant differences in susceptibility across drugs. Interestingly, these differences were more pronounced with less effective treatments (FBZ and IVM), with *G. carassii* consistently showing higher susceptibility than the other species. In fish ectoparasites, parasite localisation on the host may affect treatment efficacy. For example, copper sulphate used to treat *Ichthyophthirius multifiliis* in channel catfish was more effective against trophonts on the skin surface than those embedded within gill filaments (Schlenk et al. 1998). However, *in vitro* tests expose all parasites under identical conditions, so observed differences among closely related species must be attributed to intrinsic factors, such as variations in metabolic pathways or target molecule structures (Lacey 1989; Timson 2016). These findings underscore the importance of species-level evaluation of drug efficacy, even within a single genus.

Despite providing valuable insights into species-specific drug susceptibility, our study has some limitations. First, all experiments were conducted under *in* *vitro* conditions, which may not fully replicate the complexity of host–parasite interactions or pharmacokinetic dynamics *in* *vivo*. However, the primary aim was to compare the sensitivity of different parasite species to therapeutic baths under standardised conditions. Second, the assays were performed at relatively low water temperatures, which may have influenced parasite metabolism and drug activity, potentially limiting generalisability to warmer environments. Third, the study focused on four parasite species and three commonly used anthelmintics, restricting broader extrapolation across taxa and compound classes. Finally, this study did not consider potential temporal drug degradation, which may have affected the long-term efficacy of selected anthelmintics. Nonetheless, by focusing on survival outcomes across congeneric species, this study provides a foundation for further research. Follow-up studies incorporating *in* *vivo* trials, a broader temperature range, a positive control (e.g., NaCl), and expanded taxonomic and pharmacological coverage would help validate and extend these findings.

## Supplementary Material

Supplementary Figure 1

Supplementary Table 1
