# Supplementary material for: In vitro comparison of anthelmintic efficacy across Gyrodactylus species
Source: Vet Med (Praha). 2026 Apr 30;71(4):129–38. doi: 10.17221/79/2025-VETMED (PMC13173408; doi:10.17221/79/2025-VETMED)
Supplement: Supplementary Figure 1 [file VETMED-71-04-125079-s001.pdf]

https://doi.org/10.17221/79/2025-VETMED

# *In vitro* comparison of anthelmintic efficacy across *Gyrodactylus* species

*Marketa Ondrackova*[1]*, *Jitka Kolarova*[2], *Katerina Skocovska*[1]

[1]*Institute of Vertebrate Biology of the Czech Academy of Sciences, Brno, Czech Republic*
[2]*South Bohemian Research Centre of Aquaculture and Biodiversity of Hydrocenoses, Faculty of Fisheries and Protection of Waters, University of South Bohemia in České Budějovice, Vodňany, Czech Republic*

*Corresponding author: ondrackova@ivb.cz*

The authors are fully responsible for both the content and the formal aspects of the electronic supplementary material. No editorial adjustments were made.

**Electronic Supplementary Material (ESM)**

Figure S1. Kaplan-Meier plot comparing survival of the four gyrodactylid species

https://doi.org/10.17221/79/2025-VETMED

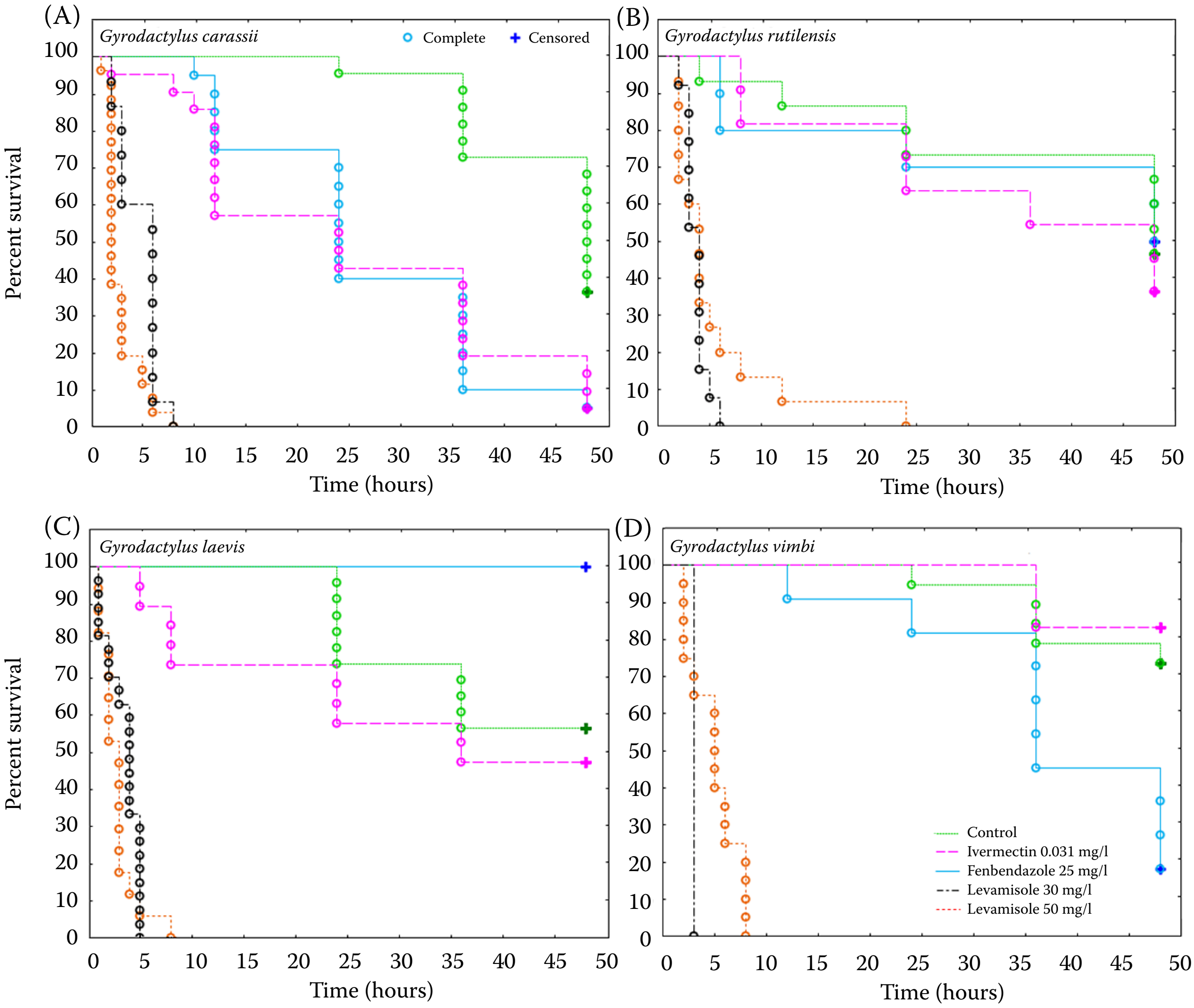


Figure S1. Kaplan-Meier plot comparing survival of the four gyrodactylid species
(A) *G. carassii*, (B) *G. rutilensis*, (C) *G. laevis*, and (D) *G. vimbi* exposed to therapeutic baths with fenbendazole 25 mg/l, levamisole 30 mg/l, and 50 mg/l, ivermectin 0.031 mg/l and control
